# Pharmacists self-perceived role competence in prevention and containment of COVID-19: A cross-sectional study

**DOI:** 10.1016/j.amsu.2021.102243

**Published:** 2021-03-26

**Authors:** Suhaib Muflih, Sayer Al-Azzam, Lynn Lafferty, Reema Karasneh, Ola Soudah, Yousef Khader

**Affiliations:** a Dept. of Clinical Pharmacy, Jordan University of Science and Technology, Jordan; b Department of Pharmacy Practice, Nova Southeastern University, USA; c Department of Basic Medical Sciences, Yarmouk University, Jordan; d Department of Public Health, Jordan University of Science and Technology, Jordan

**Keywords:** COVID-19, Pharmacists, Patient referral, Competence, Prevention, And control

## Abstract

**Introduction:**

The increased need for prioritized infection prevention and control (IPC) activities for the prevention and containment of COVID-19 is pivotal and timely in preventing harm caused by the COVID-19 pandemic. Little is known about pharmacists' infection IPC activities and their role competence during disease outbreaks. This study aimed to assess pharmacists' perceived role competence to perform frontline roles during the COVID-19 pandemic.

**Method:**

A cross-sectional survey was conducted using online social media to recruit eligible participants. A validated questionnaire contained 41 items on sociodemographic characteristics, preventative behaviors, and competencies.

**Results:**

A total of 486 participants completed the survey. Participants reported several IPC activities that could potentially prevent COVID-19 spread. The majority expressed high attitudes towards their capabilities to fulfill their healthcare roles (M = 4.43, SD = 0.46, out of 5). The vast majority of participants (97.1%) were willing to demonstrate the effective way of cleaning hands and using facemasks. Pharmacists (89.1%) showed their willingness to timely refer patients in response to their emerging needs. Gender, age groups, years of experience, monthly incomes, area of work, ability to make a referral, source of information, and self-isolation discontinuation criteria were significantly associated with pharmacists’ self-perceived role competence.

**Conclusion:**

Pharmacists are well-positioned as access points to care and can potentially play a significant role in the containment of the COVID-19 outbreak by delivering advanced clinical and public health services. Future research efforts need to be comprehensively directed towards the advanced role of pharmacists in implementing point-of-care testing for infectious diseases.

## Highlights

• Several pharmacist activities proved useful in deterring the spread of COVID-19.
• COVID-19 management is enhanced by offering pharmaceutical and public health services.
• Pharmacists demonstrated their capacity in referring patients and meet their needs.
• Pharmacists emphasized that supportive care became essential for COVID-19 patients.

## 1 Introduction

According to the World Health Organization (WHO), a newly discovered virus that belongs to the coronavirus family has led to the outbreak of the 2019 novel coronavirus diseases (COVID-19) [1]. Patients with COVID-19 generally present with mild to moderate respiratory symptoms, while older adults and those with underlying chronic diseases are expected to have a more severe course of illness [1,2]. The WHO reported that COVID-19 mainly spreads through mucus and saliva droplets that are ejected from the mouth or the nose of infected people when they cough or sneeze without adhering to protective behaviors [1]. There are currently no specific or effective treatments for COVID-19. Therefore, the best way to prevent and slow down COVID-19 transmission is to follow favorable practice recommendations (e.g., washing the hands or using an alcohol-based rub frequently, social distancing, and wearing a facemask) [1,3]. Hence, this pandemic has put many countries into lockdown to contain the disease and save lives, which will pose another challenge on governments in regards to implementing safe and effective re-opening strategies when the number of COVID-19 cases starts to plateau [4].

The expanded need for prioritized infection prevention and control (IPC) activities for the prevention and containment of COVID-19 is pivotal and timely in preventing harm caused by COVID-19. The goal of these IPC activities in the COVID-19 pandemic is to avoid COVID-19 transmission among healthcare workers (HCWs) and patients, which necessitates rapid detection of suspected cases, prompt isolation of patients, referral for testing, effective clinical management, and adherence to home isolation instructions [5]. Healthcare providers including pharmacists should be able to determine a person under investigation (PUI) (i.e., individuals with fever, cough, trouble breathing, or those who have arrived recently from areas where COVID-19 is highly spread) [6].

Pharmacists, in particular, play a pivotal role in public health by proposing effective preventative measures against infectious and non-communicable diseases, as well as supporting the uptake of vaccination [7,8]. For example, during the previous outbreak of the Ebola virus disease (EVD), pharmacists were at the front line actively participating in controlling EVD and ensuring safe and effective prescribed treatments [9]. Pharmacists also served as effective resources of information to help patients gain more insight regarding the H1N1 pandemic by initiating effective communications and conveying up-to-date health-related information [10,11].

Further, the American Society of Health-System Pharmacists (ASHP) emphasized the pivotal role of pharmacists in infection prevention and control programs in health systems [12]. As pharmacists are expected to obtain complete background information and practice evidence-based medicine to enhance decision-making [13]. In the context of the COVID-19 outbreak, attention should be called to the importance of the provision of pharmaceutical care and to the role of pharmacists in the timely detection of infected cases, effectively identifying the signs and symptoms of the disease, and answering patients' questions. Besides, pharmacists at both hospital and community levels are positioned as access points of care to maximize patients' health outcomes throughout several activities, such as patients’ education and counseling on the risks of COVID-19, and the available and effective preventive measures to limit its spread [14]. Also, the authors claimed that effective case evaluation is among those pharmaceutical activities. Competent pharmacists consider a patient referral once further consultation is needed based on many factors affecting the choice of specialists suitable for each case. When a patient is suspected of having COVID-19, pharmacists recommend further testing and evaluation to confirm or rule out the diagnosis. This enables appropriate management to be established based on the clinical and laboratory results of the case.

In response to the COVID-19 pandemic, Jordan has declared a state of emergency to curb its spread, and to prevent any severe impact on the national healthcare system [15]. The procedure was associated with the successful containment of COVID-19 at the beginning of the pandemic, where it focused on a national approach that promoted social distancing, wearing facemasks and hand washing. As of the March 2, 2020, the Prime Ministry of Jordan confirmed the first local case of COVID-19. More than 437 positive cases were recorded seven weeks after the epidemic began, with an estimated patient fatality rate of 1.60% [15]. However, the number of confirmed COVID-19 cases continued to rise sharply, and by August 31, 2020, there were over 2034 confirmed COVID-19 cases, including 15 deaths [16]. Pressure on the healthcare systems exerted by funding challenges, different perceived expectations of containment measures by the public, unavailability of vaccines, expensive testing, and miscommunication regarding the significance of COVID-19 had led to the rapid spread of the disease [17,18]. Thus, this study aimed at assessing pharmacists’ perceived role competence to perform frontline roles during the COVID-19 pandemic.

## 2 Methodology

### 2.1 Study design

A cross-sectional, web-based survey was used to recruit participants from hospital and community pharmacies in Jordan. Social media platforms (e.g., Facebook, WhatsApp) were used to recruit survey participants. A Google Form was used to collect data from eligible participants from March 10th through April 15th, 2020, via sharing the survey link on WhatsApp and Facebook professional groups (e.g., Jordan Pharmacists Association). To be included in the study, potential participants had to be part-time or full-time registered pharmacists, working in community or hospital settings, and willing to voluntarily participate in this study. Pharmacists who agreed to participate had to sign the online consent form attached to the first page of the survey in order to fill the questionnaire (Appendix A: Participation Invitation Letter). To prevent missing data and avoid duplicate responses, the validation options “Required” and “Limit to one answer” were used on Google Forms. The current study was reported in compliance with the STROCSS criteria [19]. The study was registered with the Research Registry and assigned a unique identifying number (UIN): researchregistry6575, which can be found at https://www.researchregistry.com/browse-the-registry#home/.

### 2.2 Instrument

The survey contained 41 items that assess sociodemographic characteristics, IPC activities, and pharmacists' self-perceived role competence in response to the COVID-19 pandemic. The majority of the survey items were developed after an extensive review of relevant literature and some were adapted from previously published questionnaires [9,20,21]. The questionnaire was designed to collect participants' demographics (e.g., age, gender, work setting) and socioeconomic status (e.g., monthly income). Eight items were used to assess pharmacists' self-perceived role competence in response to the COVID-19 pandemic. A single item was used to capture the best management at the current time. Three items were used to determine pharmacists' ability to guide patients to discontinue home isolation based on the criteria recommended by the Centers for Disease Control and Prevention (CDC) [22]. The 5-point Likert scale was used to measure pharmacists’ levels of agreement to the self-perceived role competence in response to COVID-19, by giving participants five responses (Strongly Disagree = 1, Disagree = 2, Neutral = 3, Agree = 4, and Strongly Agree = 5).

Participants' IPC activities were measured with 7 items written by authors following the protective guideline issued by the Jordanian government. Participants were asked to indicate how often they engaged in IPC activities since the outbreak of COVID-19 on a five-point scale (from never = 1 to very often = 5). A higher IPC score indicated that participants displayed more infection preventative measures. Four more items were added to assess pharmacists' decisions for vigilant waiting and other activities relating to the prudent use of antibiotics and herbal products as part of prevention and control measures during the COVID-19 pandemic. Four specialists in the fields of pharmacotherapy and epidemiology checked the survey items to make appropriate adjustments and ensure face and content validity. After gathering data from 15 pilot study participants who were not included in the research group, the questionnaire's reliability was established. Cronbach's alpha values for the pharmacists' self-perceived role competence and IPC items were 0.78 and 0.71, respectively. As the English language is the official language of the textbooks, teaching and learning in pharmacy schools in Jordan, this survey was developed and administered in it. (see Appendix B).

### 2.3 Ethical considerations

Ethical approval was obtained from the Institutional Review Board (IRB, Reference# 21/132/2020) at Jordan University of Science and Technology. Participants were informed about the study's anonymity and voluntary nature, as well as the confidentiality of information gathered in this study, and the risks and benefits of participation. The online survey included informed consent, and potential participants couldn't see the questions until they selected the “I agree” button, indicating their willingness to participate.

### 2.4 Statistical analysis

IBM SPSS® version 24.0 was utilized to analyze the data that were collected on Google Forms and exported to a Microsoft Excel file then imported to it. Descriptive statistics (e.g., mean (M) with standard deviation (SD)) were used to illustrate participants' characteristics and responses. Pearson correlation analysis was performed between pharmacists’ IPC activities and their self-expected role competence in response to the COVID-19 pandemic. Further, *t*-test and one-way analysis of variance (ANOVA) were conducted to investigate the association between pharmacists' self-perceived role competence and sociodemographic characteristics. While the *t*-test was applied to examine differences between the means of two groups (i.e., gender, area of living, and willingness to make a referral) on the dependent variable of interest, a one-way ANOVA was performed to examine possible relationships between means of three or more groups of the independent variables. Assumptions for T-test and one-way ANOVA were carefully assessed prior to performing statistical analysis. Raosoft® was utilized to calculate the appropriate sample size that should be included in this study. Using a significance level of 0.05 and a 5% error margin, the minimum sample size was determined to consist of 377 observations [23].

## 3 Results

A total of 486 pharmacists completed the survey during the period of the study. About three quarters (78.6%) of participants were female pharmacists. Almost one third of the participants were 25 years old or younger. Almost half (52.1%) of the pharmacists had a bachelor's degree in pharmacy and were working as full-time pharmacists in community pharmacies with a less than 5 years of working experience. The sociodemographic characteristics of participants are shown in Table 1.

**Table 1:** Frequency distribution of sociodemographic characteristics of participants (N = 486).

Variable	Frequency (%)
Gender	
Male	104 (21.4)
Female	382 (78.6)
Age group	
Up to 25	179 (36.8)
26-30	143 (29.4)
31-35	67 (13.8)
Above 35	97 (20.0)
Experience (years)	
≤5	242 (49.8)
6-10	79 (16.3)
>10	165 (34)
Levels of education	
Diplomate in Pharmacy	52 (10.7)
Bachelor of Pharmacy	253 (52.1)
PharmD	106 (21.8)
Master's Degree in Pharmacy or a higher degree	75 (15.4)
Current work situation	
Full-time Hospital Pharmacy	156 (31.8)
Full-time Community Pharmacy	197 (40.2)
Part-time in Community Pharmacy	133 (27.4)
Monthly income (USD$)	
Less than 700	274 (56.4)
700-1400	156 (32.1)
More than1400	56 (11.4)
Marital status	
Married	223 (45.5)
Single	263 (54.1)
Have children	
Yes	188 (38.6)
No	298 (61.3)
Area of work	
Urban	392 (80.7)
Rural	94 (19.3)
Source of information	
Local channels and international channels	123 (25.3)
social media	283 (58.2)
WHO website and social pages	28 (5.8)
Scientific journals	16 (3.3)
Others (e.g., workplace and colleagues, and ministry of health (MOH) website)	36 (7.4)
Frequency of use of source of information	
Daily	165 (34.0)
Weekly	264 (54.3)
Monthly	57 (11.7)

Participants displayed several IPC activities that could potentially prevent COVID-19 spread among people (see Table 2). During the COVID-19 pandemic, the majority of pharmacists in this study indicated that they apply IPC measures frequently or very often to decrease COVID-19 risk and protect their patients. In addition to the IPC practices that were reported by participating pharmacists, the majority agreed that optimizing supportive care (71.6%) was fundamental for those affected by COVID-19 due to the lack of vaccination and rational treatments against COVID-19. When participants were asked about the use of dietary supplements such as Vitamin C to prevent COVID-19 or minimize its risk, they stated that they do not routinely recommend it (33.3%). Only a few participants (23.7%) believed in the clinical benefits of using available antiviral drugs to treat COVID-19. Further, it was challenging to identify CDC criteria used to determine when to discontinue home self-isolation. While one-half of the participants in this study were able to identify two criteria, only 10% correctly identified three criteria.

**Table 2:** IPC activities, recommended treatment, and criteria for discontinuation of self-isolation for COVID-19 patients as perceived by pharmacists.

Course of actions (IPC) followed by pharmacists to minimize the spread of COVID19	Frequency (%)					Mean (SD)
	Never	Less Often	Sometimes	Often	Very Often	
Nothing, wait and see	46 (9.5)	167 (34.4)	187 (38.5)	81 (16.7)	5 (1.0)	2.65 (0.90)
Covering nose and mouth when sneezing	10 (2.1)	29 (5.7)	26 (5.3)	324 (66.7)	97 (20.0)	3.95 (0.87)
Request medical help immediately	18 (3.7)	120 (24.7)	147 (30.0)	183 (37.7)	19 (3.9)	3.14 (0.95)
Self-isolation and request sick leave	14 (2.9)	16 (3.3)	14 (2.9)	264 (54.3)	178 (36.6)	4.19 (0.87)
Take antibiotics	130 (26.7)	165 (34.0)	168 (34.6)	15 (3.1)	8 (1.6)	2.19 (0.92)
Herbal products	25 (5.1)	114 (23.5)	229 (47.1)	104 (21.4)	14 (2.9)	2.93 (0.87)
Over the counter (OTC) medications	78 (16.0)	24 (4.9)	108 (22.2)	232 (47.7)	44 (9.1)	3.29 (1.20)
Wear face mask	107 (22.0)	125 (25.7)	126 (25.9)	120 (24.7)	8 (1.6)	2.58 (1.13)
Undergo testing for COVID-19	52 (10.7)	60 (12.3)	46 (9.5)	242 (49.8)	86 (17.7)	3.51 (1.22)
Stop socializing	15 (3.1)	5 (1.0)	27 (5.6)	311 (64.0)	128 (26.3)	4.09 (0.79)
Clean surrounding surfaces	13 (2.7)	20 (4.1)	41 (8.4)	348 (71.6)	64 (13.2)	3.88 (0.78)

**Recommended treatment approach for controlling COVID-19**	**Frequency ‘Yes’ (%)**
No treatment available	398 (81.9)
Antivirals	115 (23.7)
Vitamin C	162 (33.3)
Treating complications (supportive care)	348 (71.6)
Observation and follow-up	144 (29.6)
Antibacterial	7 (1.4)

**Conditions to discontinue self-isolation of COVID 19 diagnosed patients**	**Frequency ‘Correct Answer’ (%)**
Resolution of fever with the use of fever-reducing medications (correct answer: No)	266 (54.7)
Improvement in respiratory symptoms (e.g., cough, shortness of breath) (correct answer: Yes)	232 (47.7)
Negative results of an FDA emergency use authorized molecular assay for COVID-19 from at least two consecutive nasopharyngeal swab specimens collected ≥24 h apart (correct answer: Yes)	371 (76.3)

Additionally, the IPC and pharmacists’ self-perceived role competence were significantly related (r = 0.18, n = 486, *p* < 0.05). Also, the findings of this study revealed that older age and longer duration of experience were significantly associated with higher IPC activities (r = 0.72, n = 486, *p* < 0.05; r = 0.13, n = 486, *p* < 0.05, respectively). Moreover, pharmacists who were willing to refer patients to healthcare facilities reported higher IPC activities compared to those who refused to do so (*t* (484) = 9.31, *p* < 0.05).

The mean score of attitudes toward pharmacists’ self-perceived role competence was 4.43 out of 5, with a standard deviation of 0.46. The findings showed that pharmacists agreed or strongly agreed (98.8%) about their pivotal role in instructing clienteles to keep good hand hygiene, maintain distancing behaviors, avoiding crowded places, and other infection control measures (see Table 3). More important, pharmacists (89.1%) showed their willingness to timely refer patients in response to their emerging needs. Almost 96% of the participants acknowledged their responsibility to share correct information with patients. The vast majority of participants (97.1%) were willing to demonstrate the effective way of cleaning hands and using facemasks as it is strongly believed that COVID-19 could spread through contaminated surfaces and respiratory droplets, and 95.3% were able to direct patients towards reliable sources of information if they needed to read more details about COVID-19. When participants were asked to rate their tendency to provide OTC medications to treat minor ailments such coughs, nasal congestion, runny nose, pain, and fever, 71.4% agreed or strongly agreed to do so, however, a lower percentage of participants indicated their ability to identify COVID-19 risk factors (64.4%). Nonetheless, most of the participating pharmacists (99.3%) agreed to keep themselves informed with the latest information on COVID-19.

**Table 3:** Pharmacists’ self-perceived role competence in COVID-19 disease (N = 486).

Statement	Frequency (%)					
	Strongly Disagree	Disagree	Neutral	Agree	Strongly Agree	Mean (SD)
Reminding clienteles to keep good hand hygiene, maintain distancing behaviours, avoiding crowded places, and other infection control measures.	1 (0.2)	0 (0.0)	5 (1.0)	100 (20.6)	380 (78.2)	4.77 (0.48)
Pharmacists may help expedite the referral process for patients with sign and symptoms of COVOD-19	4 (0.8)	11 (2.3)	38 (7.8)	154 (31.7)	279 (57.4)	4.43 (.74)
Pharmacists can help provide patients with necessary information about COVID-19 (e.g., signs and symptoms) to reduce visits to healthcare facilities	1 (0.2)	4 (0.8)	16 (3.3)	154 (31.7)	311 (64.0)	4.58 (0.62)
Pharmacists can advise people about the most effective way clean hands and how to correctly put on a face mask	1 (0.2)	2 (0.4)	11 (2.3)	143 (29.4)	329 (67.7)	4.64 (0.57)
Pharmacists can continue to direct clienteles to reliable resources of information such as the World Health Organization (WHO), the CDC, and local health departments	0 (0.0)	2 (0.4)	21 (4.3)	154 (31.7)	309 (63.6)	4.58 (0.59)
Pharmacists should continue to provide over-the-counter treatments for patients suffering from coughs, nasal congestion, runny nose, pain, fever, and sore throat when needed.	15 (3.1)	40 (8.2)	84 (17.3)	149 (30.7)	198 (40.7)	3.98 (0.98)
Pharmacists have the ability to identify patients who have the health risk factors for COVID-19	11 (2.3)	49 (10.1)	113 (23.3)	163 (33.5)	150 (30.9)	3.62 (0.95)
Pharmacists should remain up-to-date on the latest information so they can help provide the appropriate management for COVID-19 patients	1 (0.2)	2 (0.4)	0 (0.0)	147 (30.2)	336 (69.1)	4.68 (0.52)

The *t*-test results showed a significant effect of gender (t (484) = 2.39, p < 0.05), area of work (t (484) = 3.08, p < 0.05), and willing to make referrals (t (484) = 10.90, p < 0.05) on the overall pharmacists' self-perceived role competence scores. One-way ANOVA results also showed a significant effect of age [F (3,482) = 2.94, p < 0.05], monthly income [F (3,482) = 3.18, p < 0.05], source of information [F (4,481) = 2.76, p < 0.05], and ability to identify criteria for self-isolation discontinuation [F (3,482) = 5.20, p < 0.05] on the overall pharmacists’ self-perceived role competence scores, as shown in Table 4.

**Table 4:** The association between pharmacists’ self-perceived role competence in response to COVID-19 and sociodemographic characteristics.

	N = 486	Mean (SD)	p-value
**Gender**			
Male	104	4.54 (0.30)	0.017
Female	382	4.40 (0.29)	
**Age**			
Up to 25	179	4.39 (0.48)	0.039
26-30	143	4.42 (0.48)	
31-35	67	4.41 (0.42)	
Above 35	97	4.55 (0.44)	
**Monthly income (USD$)**			
Less than 700	274	4.41 (0.46)	0.042
JOD 700–1400	15	4.42 (0.46)	
More than 1400	56	4.58 (0.47)	
**Area of work**			
City	392	4.46 (0.44)	0.002
Rural	94	4.30 (0.51)	
**Current work situation**			
Full-time Hospital Pharmacy	156	4.37 (0.47)	0.143
Full-time Community Pharmacy	197	4.47 (0.45)	
Part-time in Community Pharmacy	133	4.42 (0.46)	
**Level of education**			
Diplomate in Pharmacy	52	4.42 (0.29)	0.666
Bachelor of Pharmacy	253	4.42 (0.31)	
PharmD	106	4.41 (0.27)	
Master's Degree in Pharmacy or a higher degree	75	4.45 (0.30)	
**Sources of information**			
Local and International Channels	123	4.47 (0.44)	0.029
Social Media	263	4.40 (0.47)	
WHO Website	28	4.51 (0.44)	
Scientific Journals	36	4.68 (0.37)	
Others (e.g., Workplace and Colleagues, and Ministry of Health (MOH) Website)	36	4.32 (0.51)	
**Number of self-isolation discontinuation criteria identified**			
Zero criteria identified	30	4.25 (0.54)	0.007
One criterion identified	93	4.31 (0.54)	
Two criteria identified	313	4.48 (0.48)	
Three criteria identified	50	4.52 (0.50)	
**Willingness to make patient referral**			
Yes	202	4.48 (0.42)	0.026
No	284	4.39 (0.49)	

Independent Samples T-test, One-way ANOVA, SD: Standard Deviation.

## 4 Discussion

To the researchers' knowledge, this is the first study that measures pharmacists' self-perceived role competence in Jordan as little is known about pharmacists' IPC activities and role competence during disease outbreaks. The findings of this study were consistent with that of a cross-sectional study conducted by Miller et al. (2012), in which 19 pharmacy students, at various community pharmacy advanced pharmacy practice experience sites across New York State, engaged in preventative behavioral responses to emergent public health concerns from September 2009 to February 2010. The authors concluded that pharmacy students were a valuable resource for patients seeking information about the H1N1 pandemic. In the time of a pandemic outbreak, this trust between patients and pharmacists would allow pharmacists to act as frontline health professionals [[8], [9], [10]]. This study revealed pharmacists’ exceptional significant role in healthcare during the COVID-19 pandemic. Furthermore, pharmacists showed a higher perception of their role competence and served as valuable resources for necessary health information about COVID-19 with clienteles, which is necessary to minimize the impact of misinformation. Older pharmacists and those with a longer duration of experience were more engaged in providing IPC; this could reflect their higher perceived value of the evidence-based model to apply preventative measures. Also, building upon years of experience may help possess advanced knowledge, awareness, competence, and skills. Pawowska et al. (2013), on the other hand, performed a nationwide cross-sectional survey study in Polish general hospitals to examine the role of the hospital pharmacist and the availability of both clinical and conventional pharmacy services to patients and medical staff. The sample included 166 hospital pharmacies out of a total of 273 hospital pharmacies. Younger pharmacists were more motivated to incorporate innovative pharmaceutical services, according to the survey [24], this discrepancy could be explained by the fact that older pharmacists in this study were more prone to have chronic diseases and, as a result, valued prevention services more highly. Furthermore, older pharmacists have more interactions with primary care doctors due to their longer experiences. This may strengthen the capacity of older pharmacists to refer patients and emphasize their role in providing pharmaceutical care services to improve patients' clinical and humanistic outcomes.

Moreover, the findings revealed that pharmacists' implications for IPC measures help them in referral decision-making if patients showed signs and symptoms of COVID-19, which could help minimize the household spread of COVID-19. As the current healthcare surveillance system relies on referrals from other providers to rapidly identify and isolate infected cases, pharmacists, therefore, can be a part of the referral practice model, which could alleviate the risk of increasing community transmission of COVID-19. This finding may highlight pharmacists’ novel role in preparedness and response for the pandemic. Despite minor differences in countries and regulations, pharmacists' roles have evolved globally, with similar perceptions of the pharmaceutical services they offer [25]. Pharmacists are trained and qualified to assist primary care physicians in fulfilling their patients' desires and needs. However, further training and educational programs are needed to enhance the referral process and make it an integral part of pharmacy practice, according to this study. Similar findings were reported by researchers in the US [26].

The findings also showed that the majority of pharmacists agreed on their role competence, which comprises behavioral components in guiding clienteles to keep good hand hygiene, maintaining social distancing behaviors, and avoiding crowded places to ensure that they protect themselves from getting COVID-19. These practices were consistent with the CDC and the WHO recommendations [1,4]. However, when the participating pharmacists were asked if dietary supplements (e.g., Vitamin C) had a role in preventing or minimizing the risks of COVID-19, they stated that they were hesitant about their role against COVID-19 and that they did not routinely recommend them.

The lower percentage of male participants in the present study might reflect the increased use of social media platforms by females. However, according to the Jordanian Pharmacists Association (JPS), the total number of Jordanian registered pharmacists is 22,667 where females account for more than two-thirds [27,28]. The findings here mirrored those of a recent cross-sectional study of 449 pharmacists in Jordan, which found that the majority of Jordanian pharmacists were adults between the ages of 26 and 35, and worked mainly in community pharmacies [29]. The empirical findings of this study showed that the average self-perceived role competence score of male pharmacists was significantly different from their counterparts. This could be explained by higher levels of commitment and motivation that male participants have in their careers, compared to female peers. Additionally, a high percentage of male participants had a higher monthly income and a longer duration of professional experience. After completing a cross-sectional survey of 103 patients in a rural area of the United Kingdom to assess their views of pharmaceutical services available at the community pharmacy, Merks et al. (2016) reported similar findings. Male pharmacists were found to be more regularly involved in promoting innovative pharmaceutical services than female pharmacists, according to the study [30]. Furthermore, the results revealed some evidence that pharmacists in rural areas were less willing than those in urban areas to play their clinical roles during the pandemic. This may be due to the fact that metropolitan customers are more likely to seek guidance and counseling from their pharmacists.

Moreover, pharmacists with higher incomes (over $1400) had a significantly higher mean self-perceived role competence compared to those with low income (less than $700), this may be due to the fact that as pharmacists' income rises, they would be able to invest in both clinical and public health activities in order to meet the demands of their patients. Previous conclusions from a literature review conducted by Agomo et al. (2018) were consistent with these findings [31]. Additionally, pharmacists who were aware of the latest updates on COVID-19 were more competent to deliver healthcare services and hence can reach greater professional fulfillment.

All part-time pharmacists in this study worked in community pharmacies, the majority of them were females with children, and they shared similar views regarding their role competence compared to their full-time peers. Notably, there were no significant differences between pharmacists who worked at community or hospital settings in their self-perceived role competence scores, which suggests that both groups considered themselves sufficiently informed and competent to deliver pharmaceutical care services during the COVID-19 pandemic. In a previous cross-sectional survey of 269 Malaysian pharmacists, it was observed that hospital pharmacists had higher perceptions and competencies of current pharmacy practice activities than their community pharmacy counterparts [32].

### 4.1 Limitations

The main limitation of this study is that an online, web-based survey was used. Thus, selection bias might have occurred as the sample consisted of pharmacists who have Internet access and use social media platforms. The findings, therefore, may not be representative of the whole population of Jordanian pharmacists. However, the results may be transferable as the research was broad and varied. This should enable pharmacists in Jordan and elsewhere to apply some of these results to similar circumstances. Due to the lack of an existing robust survey, the majority of the study survey items were developed and modified according to prior literature. However, using self-reported data may result in less nuanced responses. Social desirability may also influence the findings. As a result, the self-perceived role competence is vulnerable to self-report bias, which can lead to over-reporting in a socially acceptable manner.

## 5 Conclusion

Pharmacists can potentially play a significant role in the containment of the COVID-19 outbreak through engaging with IPC activities and delivering advanced clinical and public health services. Hospital and community pharmacists are competent to understand their patients’ needs and concerns, and subsequently convey concise up-to-date information about COVID-19 to the them. Thus, pharmacists should always be prepared to help other healthcare providers and the public to take necessary preventive measures during outbreaks, to save lives. Moreover, pharmacists can be a part of the referral practice model, due to their exceptional significant role in healthcare, which could alleviate the risk of increasing community transmission of COVID-19, as the majority of them showed the willingness to timely refer patients in response to their emerging needs. However, future research efforts need to be comprehensively directed towards the advanced role of pharmacists in implementing new treatment modalities and point-of-care diagnostic testing for early detection of communicable diseases. Further studies are also needed to assess the important health outcomes affected by successful clinical pharmacist intervention strategies.

## Ethical approval

Ethical approval was obtained from the Institutional Review Board (IRB, Reference# 21/132/2020) at Jordan University of Science and Technology.

## Please state any sources of funding for your research

This work was supported by the Deanship of Scientific Research at Jordan University of Science and Technology [grant number 172/2020]”The Deanship of Scientific Research at Jordan University of Science and Technology has no such involvement in the collection, analysis and interpretation of data, writing of the manuscript, and in the decision to submit the manuscript for publication.

## Author contribution

Lynn Lafferty: Dr. Lafferty participated in study design, interpretation of data, and in drafting, critically revising, and final approval of the manuscript, Reema Karasneh: Dr. Karasneh participated in study design, interpretation of data, and in drafting, critically revising, and final approval of the manuscript, Ola Soudah: Dr. Soudah participated in study design, creating the survey questionnaire, interpretation of data, and in drafting, critically revising, and final approval of the manuscript, Yousef Khader: Prof. Khader participated in the study design, creating the survey questionnaire, interpretation of data, also in drafting, critically revising, and final approval of the manuscript, Suhaib Muflih: Dr. Muflih served as the principal investigator (PI) and was responsible for the successful administrating and execution of the entire research project. The PI participated in creating the survey questionnaire, data collection, performing statistical analysis, summering the results, drafting, and final approval of the manuscript, Sayer Al-Azzam: Prof. Al-Azzam participated in the data analysis and interpretation, as well as in drafting, critically revising, and final approval of the manuscript.

## Conflicts of interest

The authors of this original work declare that they have all participated in the design, execution, reviewing, and analysis of the paper, and that they have approved the final version. Also, there are no conflicts of interest associated with this publication and no financial support that could have influences its reported results. This work is not under publication or consideration for publication elsewhere.

## Registration of rese

Name of the registry: The Research Registry
Unique Identifying number or registration ID: researchregistry6575
Hyperlink to your specific registration (must be publicly accessible and will be checked): https://www.researchregistry.com/browse-the-registry#home**arch Studies**

## Guarantor

The Guarantor is the one or more people who accept full responsibility for the work and/or the conduct of the study, had access to the data, and controlled the decision to publish.

## Financial Support and sponsorship

This work was supported by the Deanship of Scientific Research at 10.13039/501100004035Jordan University of Science and Technology [grant number 172/2020]”

## Data availability

The datasets generated during and/or analyzed during the current study are available from the corresponding author on reasonable request.

## Figures and Tables

**Table:** 

Please answer the following sociodemographic questions:
**Gender**:
Male
Female
**Under which age group (years) do you fall**?
Up to 25
26-30
31-35
Above 35
**Experience (years):**
≤5
6-10
>10
**Levels of education:**
Diplomate in Pharmacy
Bachelor of Pharmacy
PharmD
Master's Degree in Pharmacy or a Higher degree
**Current work situation:**
Working Part-time in Community Pharmacy
Working Part-time in Hospital Pharmacy
Full-time Community Pharmacy
Full-time Hospital Pharmacy
**Monthly income (USD$):**
Less than 700 (500 JOD)
700–1400 (500JOD-1,000JOD)
More than1400 (more than 1000 JOD)
**Marital status:**
Married
Single
**Have children:**
Yes
No
**Area of work:**
Urban
Rural
**Source of information (Please select all that apply):**
Local channels and international channels
social media
WHO website and social pages
Scientific journals
Others (e.g., workplace and colleagues, and ministry of health (MOH) website)
**Frequency of use of source of information:**
Daily
Weekly
Monthly
How frequently do you employ Evidence-Based Medicine to determine the most appropriate treatment plan for your patients?
Occasionally
Sometimes
Often
Very often
Are you willing to make an effective patient referral to a specialist during this pandemic?
Yes
No
Not Sure

Please indicate your agreement regarding the following infection prevention and control (IPC) measures against COVID-19, to prevent harm to patients and other health workers					
	Rarely	Less Often	Sometimes	Often	Very Often
Nothing, wait and see approach					
Covering nose and mouth when sneezing					
Request medical help immediately					
Self-isolation and request sick leave					
Take antibiotics					
Herbal products					
Over the counter (OTC) medications					
Wear face mask					
Undergo testing for COVID-19					
Stop socializing					
Clean surrounding surfaces					

In your opinion, please select the current treatment approach for controlling COVID-1		
- No treatment available	Yes	No
- Antivirals	Yes	No
- Vitamin C	Yes	o
- Treating complications (supportive care)	Yes	No
- Observation and follow-up	Yes	No
- Antibacterial	Yes	No

Please indicate which of the following conditions followed to discontinue self-isolation of COVID 19 diagnosed patients			
Resolution of fever with the use of fever-reducing medications	Yes	No	Not Sure
Improvement in respiratory symptoms (e.g., cough, shortness of breath)	Yes	No	Not Sure
Negative results of an FDA emergency use authorized molecular assay for COVID-19 from at least two consecutive nasopharyngeal swab specimens collected ≥24 h apart	Yes	No	Not Sure

Please indicate your level of agreement with the following activities that pharmacists are expected to perform during the COVID-19 pandemic (N = 486)					
	Strongly Disagree	Disagree	Neutral	Agree	Strongly Agree
-Reminding clienteles to keep good hand hygiene, maintain distancing behaviors, avoiding crowded places, and other infection control measures.					
-Pharmacists may help expedite the referral process for patients with sign and symptoms of COVOD-19					
-Pharmacists can help provide patients with necessary information about COVID-19 (e.g., signs and symptoms) to reduce visits to healthcare facilities					
-Pharmacists can advise people about the most effective way clean hands and how to correctly put on a face mask					
-Pharmacists can continue to direct clienteles to reliable resources of information such as the World Health Organization (WHO), the CDC, and local health departments					
-Pharmacists should continue to provide over-the-counter treatments for patients suffering from coughs, nasal congestion, runny nose, pain, fever, and sore throat when needed.					
-Pharmacists have the ability to identify patients who have the health risk factors for COVID-19					
-Pharmacists should remain up-to-date on the latest information so they can help provide the appropriate management for COVID-19 patients					

## Appendix A Participation Invitation letter

**Dear Pharmacist Colleagues**, we would like to invite you to participate in research conducted by research team at Jordan University of Science & Technology (JUST), consisting of community and hospital pharmacists. It is important for us as Frontline healthcare workers to be proactive in making a difference in the overall morbidity and mortality rates associated with 2019 Novel Coronavirus (COVID-19).

Before beginning, please review the information below and click the link at the bottom of this page.

The purpose of the study is to measure the pharmacist's infection prevention and control (IPC) activities for prevention and containment of COVID-19 and self-perceived role competence in response to COVID-19. Your participation in this study is completely voluntary and anonymous, which does not require you to provide your name or any other identifying information. Your participation will contribute to a better understanding of the role that pharmacists are playing during the COVID-19 pandemic. All responses will be kept confidential.

You can take this survey conveniently using your cell phone or personal computer. The survey will close on April 15, 2020.

If you agree to be in this study, you will be asked to complete a brief demographic question that will take approximately 1 min to complete. You will be asked to provide Yes/No answers and indicate your level of agreement about IPC activities and role competency during the pandemic that will take approximately 7 min to complete.

For general questions or technical issues please email the author smmuflih@just.edu.jo.
